# Supplementary material for: Ex Vivo Model of Neuroblastoma Plasticity
Source: Cancers (Basel). 2023 Feb 17;15(4):1274. doi: 10.3390/cancers15041274 (PMC9954615; doi:10.3390/cancers15041274)
Supplement: Supplementary file 1 [file cancers-15-01274-s001.zip › cancers-2213362-supplementary.pdf]

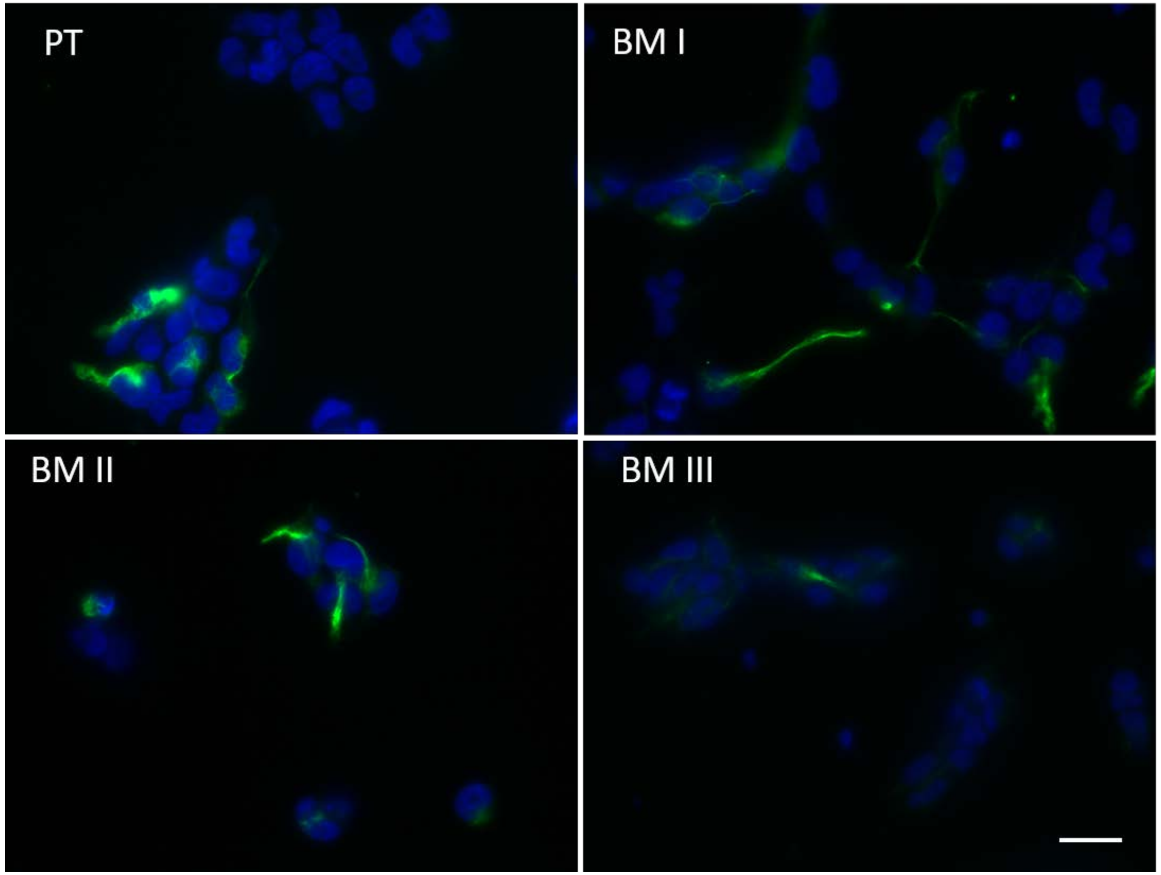

Figure S1: NFL-staining in NB-cells. Cells were treated with an Alexa-fluor488-coupled antibody against NFL (green) and the nuclei were stained with DAPI (blue). Shown is one representative image. Bar: 10 µM

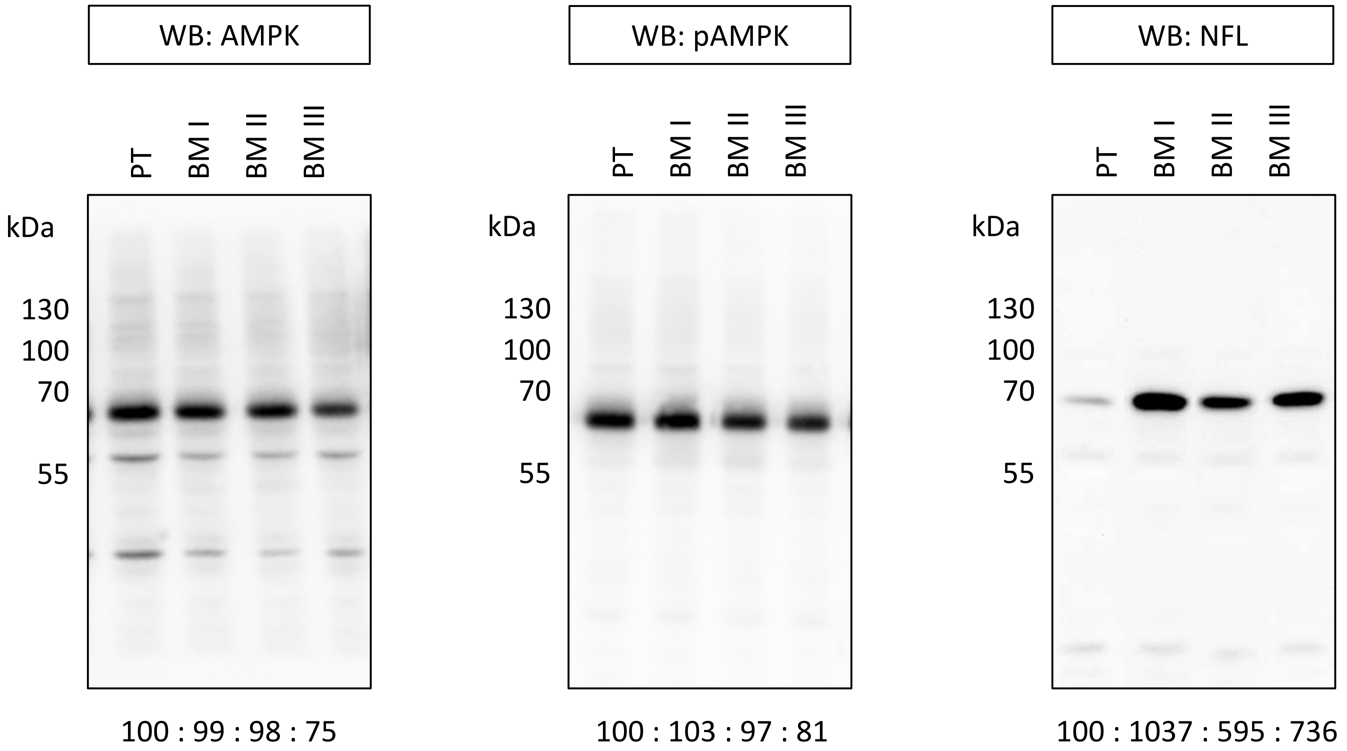

Figure S2. Original Western-blots. 20 µg protein extracts from NB cells were probed against AMPK, pAMPK or NFL by Western-blotting. Band intensities were measured by Image J, and the intensitities obtained for the PT signals were set to 100%. Show is one representative Western-blot each out of three.

**Pathway**	**PT vs BM I**	**PT vs BM II**	**PT vs BM III**
MAPK<br>MAPK signaling pathway (Homo sapiens) - WikiPathways	up: 3, down: 2<br>p = 0.84	up: 7, down: 0<br>p = 0.85	up: 4, down: 2<br>p = 0.83
PI3K/Akt<br>PI3K-Akt signaling pathway (Homo sapiens) - WikiPathways	up: 4, down: 0<br>p = 0.16	up: 2, down 1<br>**p = 0.022***	no changes
JAK/STAT<br>JAK/STAT pathway (Homo sapiens) - WikiPathways	up: 0, down: 4<br>p = 0.35	up: 0, down: 1<br>p = 0.38	no changes
Wnt<br>Wnt signaling (Homo sapiens) - WikiPathways	up: 1, down: 1	up:1; down: 0<br>p = 0.27	up: 1, down: 1
TGF-beta<br>TGF-beta signaling pathway (Homo sapiens) - WikiPathways	up: 1, down: 0<br>p = 0.27	Up:1, down: 1<br>p = 0.45	no changes
VEGF<br>VEGFA-VEGFR2 signaling pathway (Homo sapiens) - WikiPathways	up: 0, down: 1<br>**p = 0.0003***	up: 3, down: 2<br>**p = 0.022***	Up: 4; down: 1<br>p = 0.14
EGF<br>EGF/EGFR signaling pathway (Homo sapiens) - WikiPathways	up: 1, down: 1<br>p = 0.45	up: 1, down: 2<br>p = 0.64	no changes
Integrin signaling<br>Integrin signaling (Homo sapiens) - WikiPathways	up: 1, down 0	no changes	no changes
Hedgehog<br>Hedgehog signaling pathway (Homo sapiens) - WikiPathways	no changes	no changes	no changes
Hippo<br>Hippo-Merlin signaling dysregulation (Homo sapiens) - WikiPathways	up: 2, down: 1	up: 1, down 0<br>p = 0.53	no changes
Notch<br>Notch signaling (Homo sapiens) - WikiPathways	no changes	no changes	no changes
Toll-like receptor<br>Toll-like receptor signaling pathway (Homo sapiens) - WikiPathways	up: 2, down: 0	no changes	no changes
RHO GTPase signaling	no changes	no changes	no changes

Signaling by Rho GTPases (Homo sapiens) - WikiPathways			
GPCRs<br><br>GPCRs. class A rhodopsin-like (Homo sapiens) - WikiPathways	up: 3, down: 1<br><br>p = 0.42	up: 3, down: 5<br><br>p = 0.85	up = 3 down 2
Erythropoietin signalling<br><br>Signaling by Erythropoietin (Homo sapiens) - WikiPathways	no changes	no changes	no changes
